# Inhibition of RIPK1/RIPK3-MLKL inflammatory signaling pathway activation attenuates preterm birth

**DOI:** 10.1038/s41420-026-03093-z

**Published:** 2026-04-18

**Authors:** Xuexiang Bing, Yongqing Wang, Jiacui Zheng, Guodong Gao, Jinxiao Jiang, Lanlan Liu, Xue Zhang

**Affiliations:** People’s Hospital of Rizhao, Rizhao City, Shandong Province China

**Keywords:** Inflammation, Preclinical research

## Abstract

Preterm birth (PTB) is a principal contributor to neonatal morbidity, wherein inflammation and dysregulated cell death pathways are implicated as key drivers in its pathogenesis. However, the role of the RIPK1/RIPK3-MLKL signaling axis, a critical regulator of necroptosis and inflammatory responses, remains poorly characterized in the context of PTB. Here, we sought to elucidate the role of RIPK1-mediated activation of the RIPK3-MLKL pathway in placental inflammation and its involvement in PTB pathogenesis. In vitro experiments were conducted using TNF-α-stimulated HTR8/SVneo trophoblasts, while an LPS-induced murine model was employed to mimic inflammation-associated PTB. RIPK1 expression was modulated via shRNA-mediated knockdown or pharmacological inhibition with GSK2982772 and Nec-1. Molecular analyses included qPCR, Western blotting, ELISA, and the assessment of necroptosis via PI staining. We found that TNF-α and LPS significantly upregulated RIPK1 expression and activated the RIPK3-MLKL pathway in both the cellular and animal models. RIPK1 knockdown or pharmacological inhibition attenuated TNF-α-induced proinflammatory cytokine release (IL-1β, IL-6, TNF-α), uric acid accumulation, RIPK3-MLKL pathway activation, and necroptosis in trophoblasts at both 24 and 48 h. Notably, in vivo treatment with Nec-1 ameliorated LPS-induced placental damage. Collectively, our findings demonstrate that RIPK1 drives inflammation and necroptosis in PTB through RIPK3-MLKL activation, suggesting that targeting RIPK1 may represent a promising therapeutic strategy for inflammation-associated preterm labor.

## Introduction

Preterm birth (PTB), defined as delivery before 37 weeks of gestation, remains a principal driver of neonatal morbidity and mortality worldwide [1]. Approximately 11% of all live births are preterm, contributing to nearly 1 million neonatal deaths annually and long-term developmental impairments in survivors [2]. The etiology of PTB is multifactorial, with infection and inflammation identified as major contributors [3]. Inflammatory responses in the placenta and fetal membranes trigger the release of proinflammatory cytokines (TNF-α, IL-1β, IL-6), which in turn leads to premature activation of labor pathways [4]. Despite advances in obstetric care, effective therapeutic strategies to prevent inflammation-driven PTB remain insufficient, highlighting an urgent need to elucidate the molecular mechanisms underlying placental inflammation.

Receptor-interacting protein kinase 1 (RIPK1) is a critical mediator of inflammatory signaling, cell death, and innate immune responses [5]. RIPK1 acts as a crucial signaling scaffold that modulates apoptosis, necroptosis (programmed necrosis), and NF-κB-mediated inflammatory signaling [6]. Under physiological conditions, RIPK1 promotes cell survival by activating pro-survival pathways. However, in response to pathological stress such as TNF-α stimulation, RIPK1 can initiate necroptosis through its interaction with RIPK3 and mixed lineage kinase domain-like protein (MLKL) [7]. Subsequent phosphorylation of RIPK3 and MLKL leads to plasma membrane rupture, the release of damage-associated molecular patterns (DAMPs), and the amplification of inflammatory cascades [8]. Dysregulation of the RIPK1/RIPK3-MLKL axis has been implicated in various pathologies, including chronic inflammatory diseases [9], neurodegenerative disorders [10], and sepsis [11]. Intriguingly, emerging evidence implicates dysregulated cell death, including necroptosis, in placental dysfunction and adverse pregnancy outcomes [12], yet the specific contribution of the RIPK1/RIPK3-MLKL axis in the pathogenesis of inflammation-driven PTB has not been directly investigated.

Emerging evidence suggests that placental trophoblast dysfunction contributes to PTB pathogenesis [13]. Excessive trophoblast necroptosis and inflammation may disrupt placental homeostasis, leading to premature labor [14]. Given the central role of RIPK1 at the crossroads of inflammation and necroptosis, and the documented elevation of TNF-α in PTB [15] which is a potent activator of RIPK1-dependent pathways [16], we hypothesized that RIPK1-mediated activation of the RIPK3-MLKL pathway drives placental inflammation and necroptosis, ultimately promoting PTB. Targeting this signaling axis may therefore represent a novel therapeutic strategy to mitigate inflammation-associated preterm labor.

In this study, we investigated the role of RIPK1/RIPK3-MLKL signaling in PTB by employing both in vitro (TNF-α-stimulated trophoblasts) and in vivo (LPS-induced preterm mouse model) models. We further evaluated the therapeutic potential of RIPK1 inhibition via genetic knockdown and pharmacological blockade using inhibitors [17] (GSK2982772 and Necrostatin-1). Our findings provide new insights into the molecular mechanisms of PTB and highlight RIPK1 as a promising target for intervention.

## Results

### Activation of the RIPK1/RIPK3-MLKL inflammatory signaling pathway in preterm birth

**Fig. 1:**
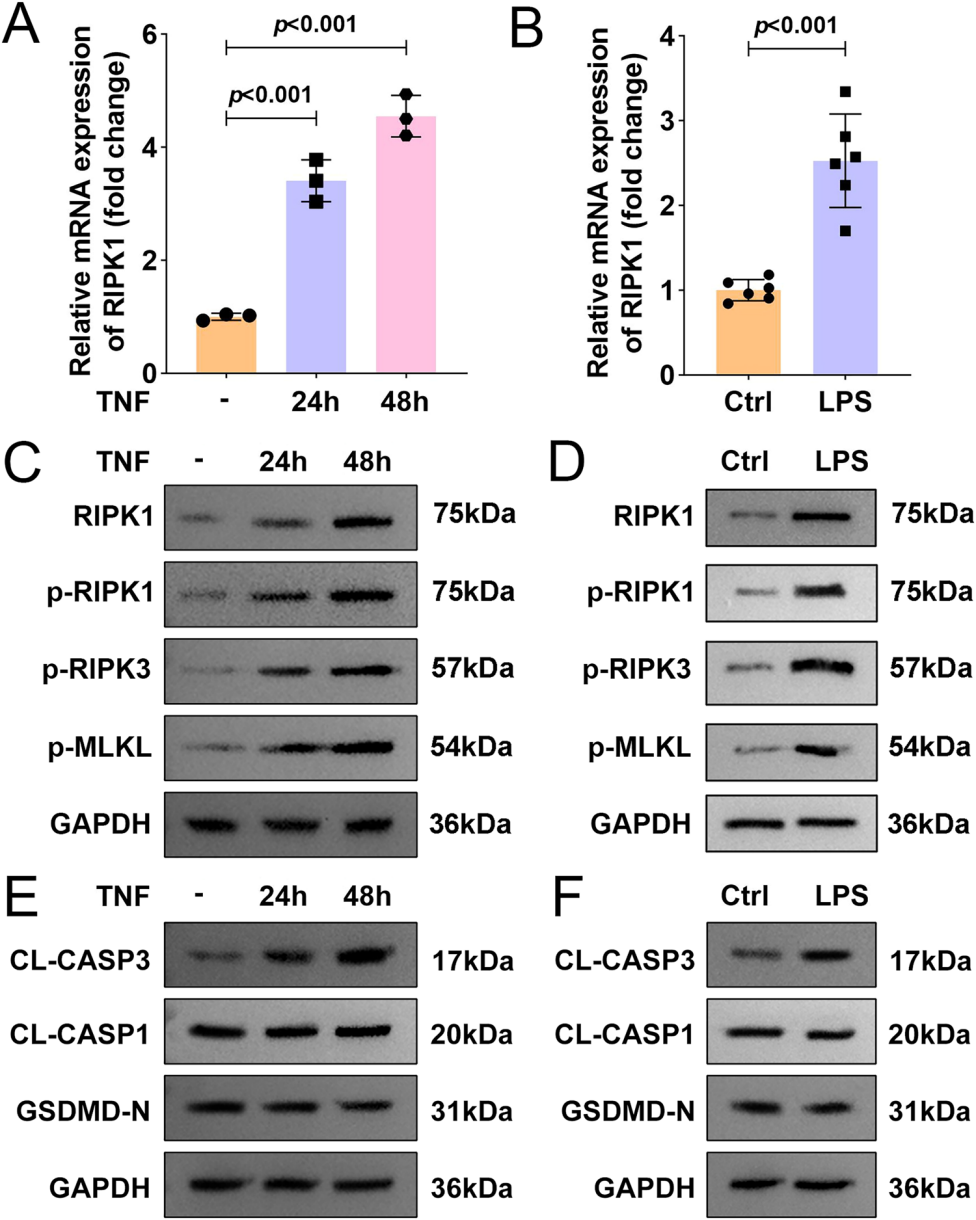
Activation of the RIPK1/RIPK3-MLKL inflammatory signaling pathway in preterm birth. **A**, **B** The mRNA level of RIPK1 were measured in TNF-α-treated HTR8/SVneo cells (*n* = 3) and placental tissues from preterm mice (*n* = 6) by qPCR. **C**, **D** The protein expression of RIPK1/RIPK3-MLKL inflammatory signaling pathway associated proteins were measured by western blot in TNF-α-treated HTR8/SVneo cells (*n* = 3) and placental tissues from preterm mice (*n* = 6). **E**, **F** The protein expression of CL-CASP3, CL-CASP1, and GSDMD-N were measured by western blot in TNF-α-treated HTR8/SVneo cells (*n* = 3) and placental tissues from preterm mice (*n* = 6). Data were analyzed by unpaired Student’s *t*-test (for two-group comparisons) or one-way ANOVA followed by Tukey’s test (for multi-group comparisons). All data are expressed as the means ± SD.

To investigate the role of RIPK1-mediated inflammation in preterm birth, we first assessed its expression in relevant models. In an in vitro model using TNF-α-stimulated HTR8/SVneo trophoblast cells, a significant upregulation of RIPK1 mRNA levels was observed at both 24 and 48 h post-stimulation (Fig. 1A). Similarly, in a murine model of LPS-induced preterm birth, RIPK1 mRNA expression was markedly elevated in placental tissues from preterm mice (Fig. 1B). Given that RIPK1 is a crucial mediator of TNFR1 signaling, we next examined the activation of the downstream RIPK1/RIPK3-MLKL inflammatory cascade. Western blot analysis revealed a robust increase in the expression of RIPK1 and the phosphorylation levels of RIPK1 (p-RIPK1), RIPK3 (p-RIPK3), and MLKL (p-MLKL) in TNF-α-stimulated human placental trophoblasts at both 24 and 48 h, as well as in placental tissues from preterm mice (Fig. 1C, D). To further explore the potential contribution of other forms of programmed cell death, we assessed the expression of key apoptosis and pyroptosis markers. As shown in Fig. 1E, the protein levels of cleaved caspase-3 (CL-CASP3) were moderately increased in TNF-α-treated trophoblasts at 24 and 48 h, whereas the pyroptosis-related markers cleaved caspase-1 (CL-CASP1) and GSDMD-N showed no significant changes. Consistent with these in vitro findings, placental tissues from LPS-induced preterm mice also exhibited elevated CL-CASP3 levels, but no notable alterations in CL-CASP1 or GSDMD-N expression (Fig. 1F). Collectively, these findings demonstrate that the RIPK1/RIPK3-MLKL inflammatory signaling pathway is activated in our preterm birth models, whereas pyroptosis does not appear to be prominently involved. RIPK1 thus emerges as a pivotal driver of inflammatory responses in both in vitro and in vivo settings, with a partial contribution from apoptotic signaling.

### The knockdown of RIPK1 attenuates TNF-α-induced proinflammatory responses in HTR8/SVneo cells

**Fig. 2:**
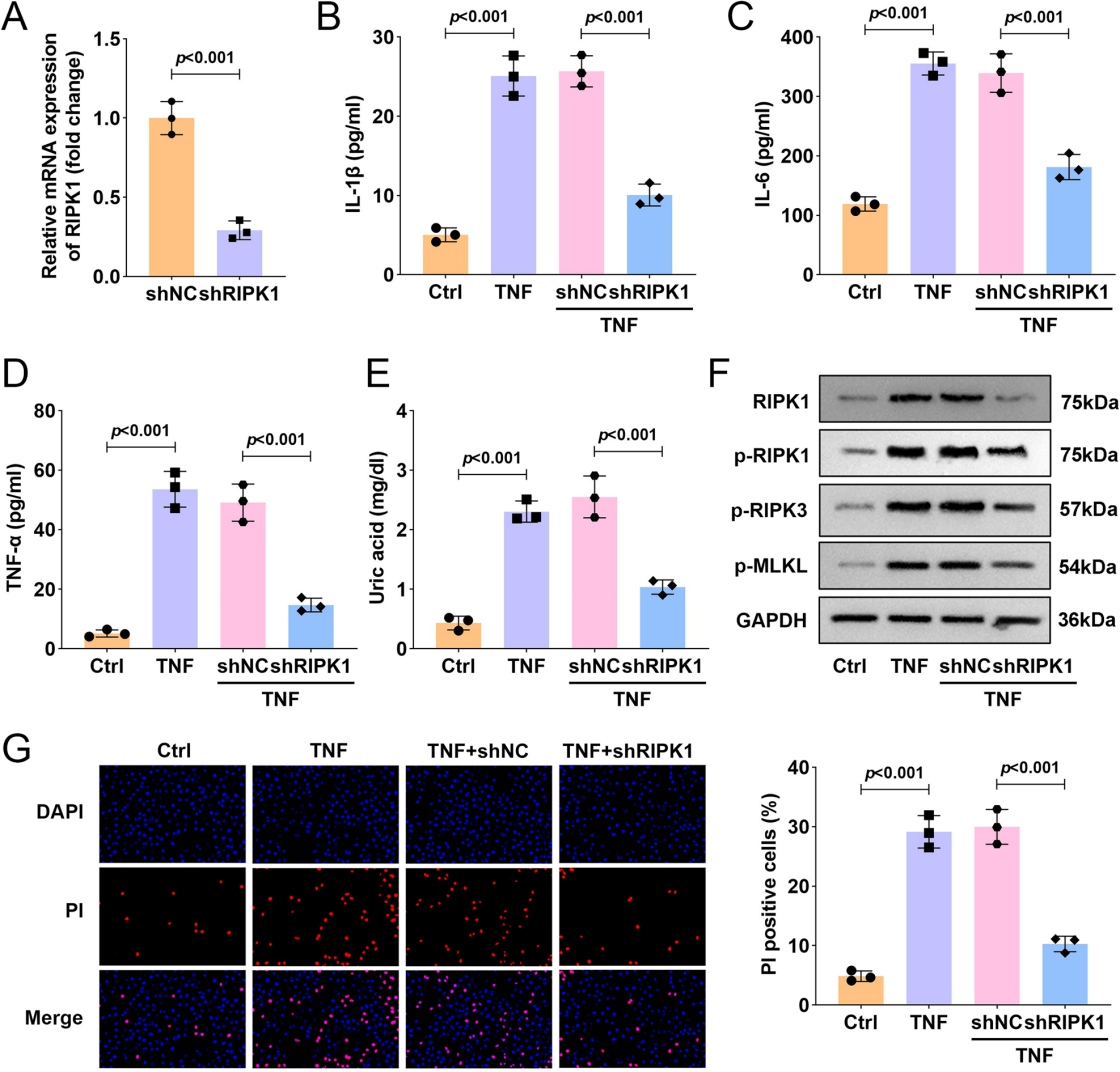
The knockdown of RIPK1 attenuated proinflammatory responses in HTR8/SVneo cells following 24-h TNF-α induction. **A** RIPK1 mRNA level detection on HTR8/SVneo cells, respectively, transfected with shRIPK1 or shNC plasmids. HTR8/SVneo cells were transfected with shRIPK1 or shNC (negative control) plasmids and then stimulated with TNF-α for 24 h. **B**–**D** The contents of IL-1β, IL-6, and TNF-α were measured by ELISA assay. **E** Uric acid concentration was measured with an assay kit. **F** The protein expression of RIPK1/RIPK3-MLKL inflammatory signaling pathway associated proteins were measured by western blot. **G** PI staining was used to determine HTR8/SVneo trophoblast cell death. Data were analyzed by one-way ANOVA followed by Tukey’s test. All data are expressed as the means ± SD. (*n* = 3).

To elucidate the functional role of RIPK1, its expression in HTR8/SVneo cells was silenced using shRNA (shRIPK1), with knockdown efficiency confirmed by qPCR after 24 h (Fig. 2A). ELISA measurements showed that TNF-α stimulation significantly increased the secretion of proinflammatory cytokines (IL-1β, IL-6, and TNF-α), which was markedly reversed by RIPK1 silencing at this 24-h time point (Fig. 2B–D). Furthermore, we assessed intracellular levels of uric acid, a key inflammatory mediator, and observed that RIPK1 knockdown suppressed the TNF-α-induced accumulation of uric acid (Fig. 2E). Western blot analysis demonstrated that RIPK1 depletion markedly inhibited the TNF-α-induced activation of the RIPK1/RIPK3-MLKL signaling pathway (Fig. 2F). Additionally, PI staining showed that TNF-α promoted necroptosis in HTR8/SVneo cells, an effect that was mitigated by RIPK1 knockdown (Fig. 2G). To substantiate the robustness of these findings, we repeated the entire set of functional assays at a 48-h time point. The results were highly consistent, showing that RIPK1 knockdown similarly attenuated the TNF-α-induced release of proinflammatory cytokines (Supplementary Fig. 1A–C), the accumulation of uric acid (Supplementary Fig. 1D), the activation of the RIPK1/RIPK3–MLKL pathway (Supplementary Fig. 1E), and the incidence of necroptosis (Supplementary Fig. 1F). Collectively, these results from both 24-h and 48-h time points indicate that RIPK1 plays a critical role in mediating TNF-α-induced proinflammatory responses and necroptosis in placental trophoblasts. Inhibition of RIPK1 attenuates inflammatory cytokine release, uric acid accumulation, and RIPK3–MLKL pathway activation, underscoring its potential as a therapeutic target in inflammation-associated preterm birth.

### GSK2982772-mediated RIPK1 inhibition effectively mitigates TNF-α-driven inflammatory responses in placental trophoblasts

**Fig. 3:**
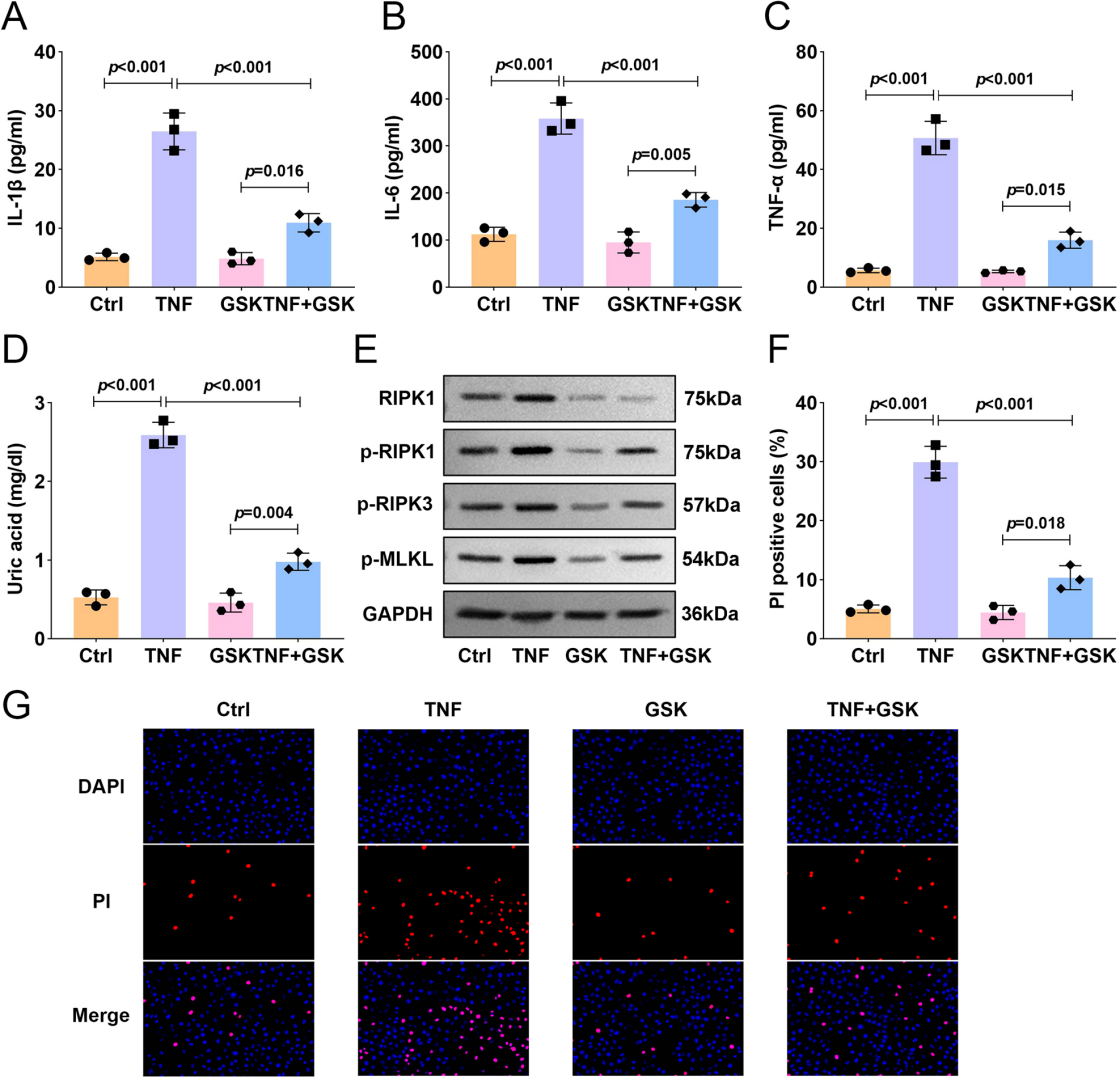
GSK2982772-mediated RIPK1 inhibition effectively mitigated TNF-α-driven inflammatory responses in placental trophoblasts. HTR8/SVneo trophoblast cells were stimulated with TNF-α after treatment with or without GSK2982772. **A**–**C** The contents of IL-1β, IL-6, and TNF-α were measured by ELISA assay. **D** Uric acid concentration was measured with an assay kit. **E** The protein expression of RIPK1/RIPK3-MLKL inflammatory signaling pathway associated proteins were measured by western blot. **F**, **G** PI staining was used to determine HTR8/SVneo trophoblast cell death. Data were analyzed by one-way ANOVA followed by Tukey’s test. All data are expressed as the means ± SD. (*n* = 3).

To investigate the therapeutic potential of targeting RIPK1, we first employed GSK2982772, a highly selective RIPK1 inhibitor. Treatment with GSK2982772 effectively reversed the TNF-α-induced upregulation of proinflammatory cytokines (IL-1β, IL-6, and TNF-α) and suppressed elevated uric acid levels in HTR8/SVneo cells (Fig. 3A–D). Furthermore, while TNF-α robustly activated the RIPK1/RIPK3-MLKL signaling pathway, pharmacological inhibition of RIPK1 with GSK2982772 markedly suppressed this activation (Fig. 3E). In addition, TNF-α stimulation significantly increased the proportion of PI-positive cells, indicating enhanced necroptosis, whereas GSK2982772 treatment markedly attenuated this effect (Fig. 3F, G). These findings demonstrated that GSK2982772-mediated RIPK1 inhibition effectively mitigated TNF-α-driven inflammatory responses, uric acid accumulation, and necroptotic cell death in placental trophoblasts. These data thus support RIPK1 as a key therapeutic target for mitigating inflammation-associated preterm birth.

### Inhibition of RIPK1 by Nec-1 effectively prevents TNF-α-triggered inflammatory responses in placental trophoblasts

**Fig. 4:**
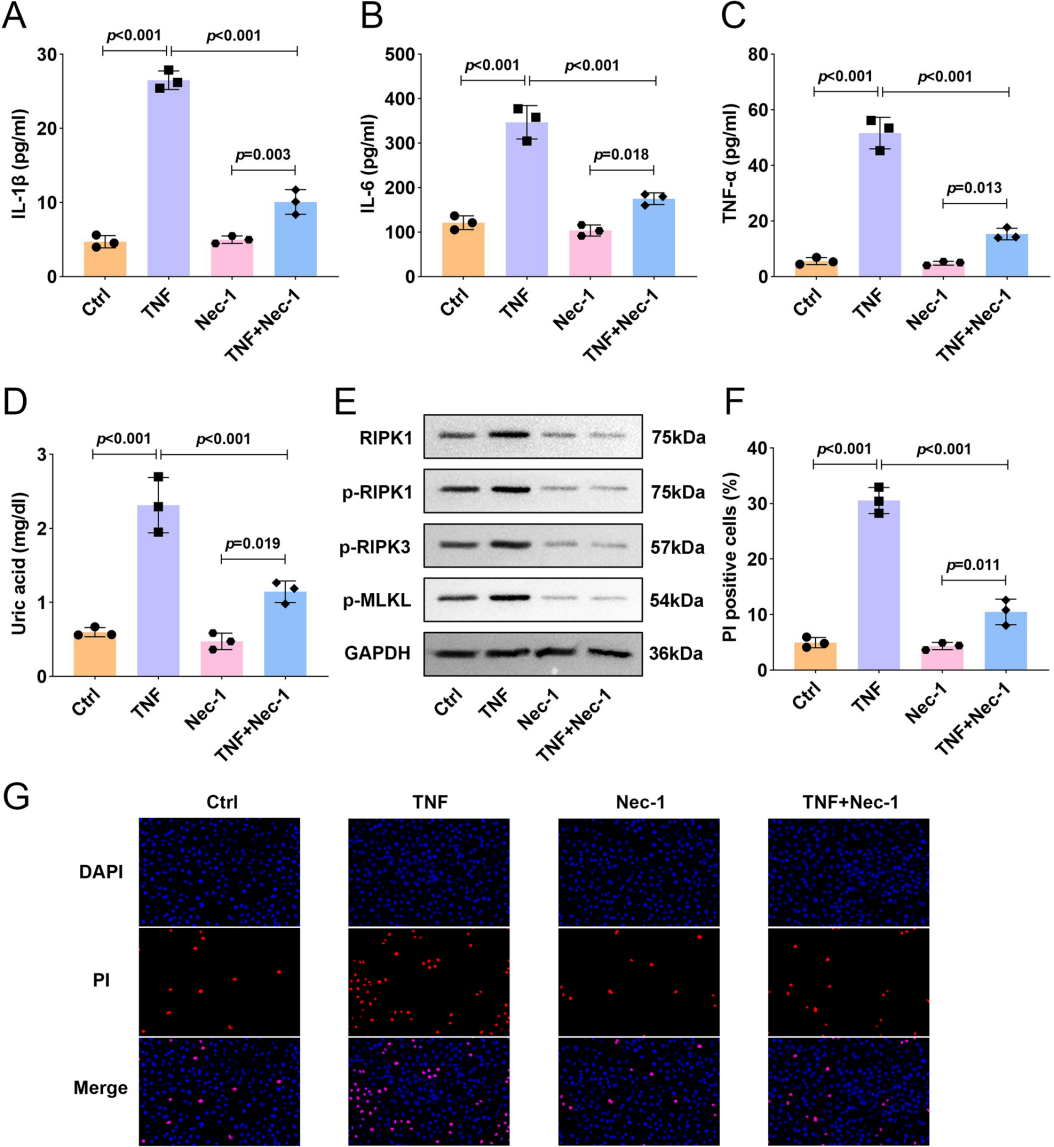
Inhibition of RIPK1 by Nec-1 effectively prevented TNF-α-triggered inflammatory responses in placental trophoblasts. HTR8/SVneo trophoblast cells were stimulated with TNF-α after treatment with or without Nec-1. **A**–**C** The contents of IL-1β, IL-6, and TNF-α were measured by ELISA assay. **D** Uric acid concentration was measured with an assay kit. **E** The protein expression of RIPK1/RIPK3-MLKL inflammatory signaling pathway associated proteins were measured by western blot. **F**, **G** PI staining was used to determine HTR8/SVneo trophoblast cell death. Data were analyzed by one-way ANOVA followed by Tukey’s test. All data are expressed as the means ± SD. (*n* = 3).

To further validate the role of RIPK1, we next utilized Necrostatin-1 (Nec-1), a potent and selective allosteric inhibitor of RIPK1. Nec-1 treatment effectively abrogated the TNF-α-induced elevation of proinflammatory cytokines (IL-1β, IL-6, and TNF-α) and normalized uric acid levels in HTR8/SVneo cells (Fig. 4A–D). Moreover, Nec-1 administration completely suppressed the TNF-α-mediated activation of the RIPK1/RIPK3-MLKL signaling axis (Fig. 4E). Complementary to these findings, while TNF-α challenge markedly increased PI-positive cell populations, Nec-1 pretreatment substantially mitigated this cytotoxic effect (Fig. 4F, G). These data demonstrate that pharmacological inhibition of RIPK1 by Nec-1 not only prevents TNF-α-triggered necroptosis but also disrupts the subsequent inflammatory cascade in placental trophoblasts.

### Inhibition of RIPK1 by Nec-1 effectively mitigates LPS-mediated tissue damage in preterm birth murine model

**Fig. 5:**
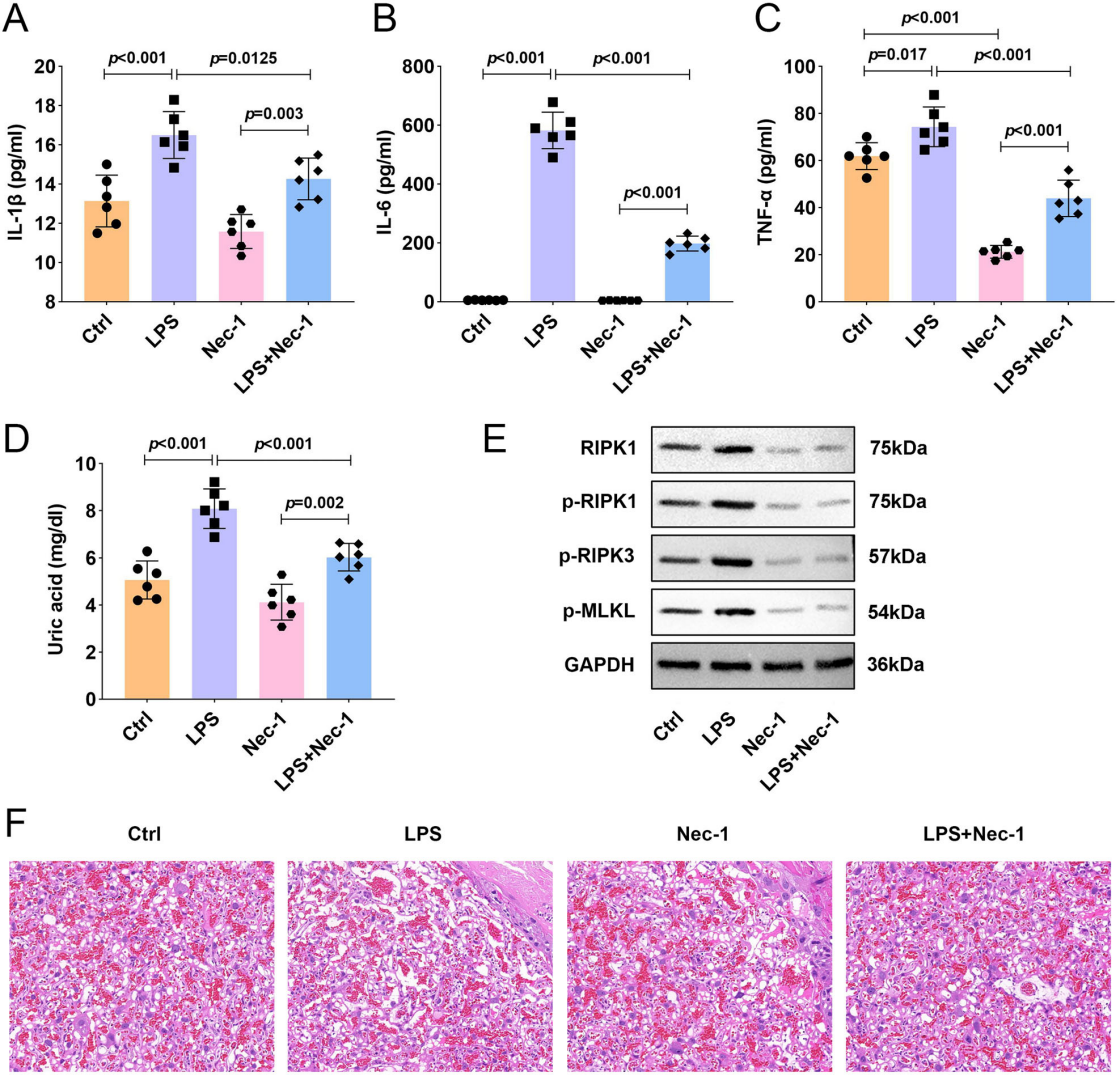
Inhibition of RIPK1 by Nec-1 effectively mitigated LPS-mediated tissue damage in preterm birth murine model. Mice were intraperitoneally injected with or without 0.5 mg/kg LPS and treated with or without 0.5 mg/kg Nec-1. **A**–**C** The contents of IL-1β, IL-6, and TNF-α were measured by ELISA assay. **D** Uric acid concentration was measured with an assay kit. **E** The protein expression of RIPK1/RIPK3-MLKL inflammatory signaling pathway associated proteins were measured by western blot. **F** HE staining of placental tissue. Data were analyzed by one-way ANOVA followed by Tukey’s test. All data are expressed as the means ± SD. (*n* = 6).

To assess the in vivo efficacy of RIPK1 inhibition, we administered Nec-1 in our LPS-induced murine model of preterm birth. We observed marked elevations in proinflammatory cytokines (IL-1β, IL-6, and TNF-α) and uric acid levels in response to LPS, effects that were partially but significantly reversed by Nec-1 treatment (Fig. 5A–D). Consistent with these findings, Nec-1 administration prevented the LPS-induced activation of the RIPK1/RIPK3-MLKL signaling pathway (Fig. 5E). Histopathological examination revealed that LPS challenge triggered characteristic inflammatory damage in placental tissues, including leukocyte infiltration, tissue disorganization, and nuclear abnormalities. Importantly, Nec-1 treatment substantially ameliorated these pathological changes, as evidenced by reduced inflammatory infiltration, restored tissue architecture, and normalized nuclear morphology (Fig. 5F). These results demonstrated that pharmacological inhibition of RIPK1 by Nec-1 not only attenuated systemic inflammation but also preserved placental tissue integrity during LPS-induced preterm birth.

## Discussion

Inflammation-associated PTB is characterized by excessive activation of inflammatory pathways in maternal-fetal tissues, leading to premature initiation of labor [18]. Placental trophoblasts play a central role in this process by responding to inflammatory stimuli through the production of cytokines and activation of cell death pathways [19]. In addition, necroptosis has recently emerged as a key contributor to placental dysfunction and labor initiation [12]. RIPK1 serves as a master regulator at the intersection of inflammation and cell death. Under pathological conditions, such as those induced by TNF-α stimulation, RIPK1 can initiate necroptosis through formation of the necrosome complex with RIPK3 and MLKL. This process releases DAMPs that amplify inflammatory responses, thereby creating a vicious cycle of inflammation and tissue damage [20]. In the present study, we demonstrated that RIPK1 expression was significantly upregulated in both TNF-α-stimulated human trophoblasts and LPS-induced preterm mouse placentas, suggesting that RIPK1 activation may be a common feature of inflammation-driven PTB. The concomitant increase in the phosphorylation of RIPK1, RIPK3, and MLKL in both models provides direct evidence for the activation of this pathway. Notably, both the total protein level and phosphorylation of RIPK1 were concurrently elevated following stimulation, which may reflect cell type-specific regulatory mechanisms involving transcriptional upregulation alongside kinase activation. Furthermore, the activation of this pathway was accompanied by an increase in apoptosis, but not in pyroptosis, underscoring its significance in inflammation-induced preterm birth.

The functional significance of RIPK1 upregulation was further elucidated through our knockdown experiments. Silencing RIPK1 expression in HTR8/SVneo cells markedly attenuated TNF-α-induced proinflammatory cytokine release, uric acid accumulation, and RIPK3-MLKL pathway activation in trophoblasts at both 24 and 48 h. These results suggest that RIPK1 not only regulates cytokine production but also influences the metabolic changes associated with inflammation. While RIPK1, a key kinase regulating necroptosis, is known to promote cell death and inflammation in a variety of diseases [21], our results now elucidate its specific role in PTB pathogenesis. The significant increase in PI-positive cells following TNF-α treatment, which was reversed by RIPK1 knockdown, provides compelling evidence that RIPK1 promotes necroptotic cell death in trophoblasts. This finding has important implications for understanding placental dysfunction in PTB, as excessive trophoblast death could compromise placental barrier function and trigger labor initiation [22]. The simultaneous reduction in both necroptosis and inflammation upon RIPK1 inhibition suggests that these processes are mechanistically linked in the context of PTB, with RIPK1 serving as their common regulator.

The therapeutic potential of targeting RIPK1 was further substantiated by our pharmacological studies. As a selective RIPK1 inhibitor [23], GSK2982772 has been shown to effectively attenuate tau-induced astrocytic inflammation [24] and ameliorate colitis by suppressing chemokine/adhesion molecule release and oxidative stress in damaged intestinal epithelial cells [25]. In the present study, GSK2982772 effectively mitigated all measured aspects of TNF-α-induced inflammation in trophoblasts, mirroring the effects of genetic RIPK1 knockdown. This consistency between genetic and pharmacological approaches strengthens the validity of our findings and supports the translational potential of RIPK1 inhibition.

Nec-1, a widely used small-molecule inhibitor of RIPK1, demonstrates broad protective effects across various disease models [26, 27]. For instance, it preserves corneal epithelial cells by blocking the RIPK1/RIPK3/MLKL cascade in benzalkonium chloride-induced necroptosis [28] and mitigates cerebral ischemic injury via suppression of RIPK1-dependent RIPK3/MLKL activation [29]. Additionally, Nec-1 attenuates LPS-driven inflammatory responses [30]. Our investigations with Nec-1 yielded consistent and mechanistically informative results. As an allosteric inhibitor that stabilizes RIPK1 in an inactive conformation, Nec-1 showed exceptional potency in suppressing TNF-α-induced responses. The complete suppression of TNF-α-mediated RIPK1/RIPK3-MLKL activation by Nec-1 treatment demonstrates the effectiveness of this inhibitor in blocking the entire signaling cascade. This observation that Nec-1 normalized uric acid levels is particularly relevant, as elevated uric acid has been associated with poor pregnancy outcomes [31].

These in vitro findings were corroborated by our in vivo studies, where Nec-1 administration not only suppressed systemic inflammation, as evidenced by decreased cytokine levels, but also preserved placental tissue architecture. The histological improvements, including reduced leukocyte infiltration and restoration of normal nuclear morphology, suggest that RIPK1 inhibition could protect against the structural damage that often accompanies inflammation-induced PTB. The consistent protective effects achieved through two distinct pharmacological strategies—GSK2982772 and Nec-1—alongside genetic knockdown, validate RIPK1 as a critical node in trophoblast inflammation and death. Importantly, the attenuation of uric acid accumulation, a clinically relevant marker associated with adverse pregnancy outcomes, highlights a potentially translatable metabolic benefit of RIPK1 inhibition beyond cytokine suppression.

Despite the compelling evidence presented, this study has several limitations that warrant consideration. First, our findings rely heavily on pharmacological inhibition and shRNA-mediated knockdown of RIPK1, and we did not employ in vivo genetic validation models such as RIPK1 K45A kinase-dead, RIPK3 knockout, or MLKL knockout mice. Such models would provide more definitive evidence regarding the specific contributions of RIPK1 kinase activity and the RIPK3-MLKL axis to PTB pathogenesis. Second, our analysis was primarily confined to placental tissue. Future investigations examining other maternal-fetal interface tissues, such as the decidua and fetal membranes, as well as systemic maternal organs, would be valuable to determine the broader extent of pathway activation and its systemic implications. Third, while we focused on necroptosis and briefly assessed apoptosis and pyroptosis, the potential crosstalk and compensatory mechanisms between different programmed cell death pathways in the context of PTB remain incompletely characterized. Finally, our in vivo model utilized an LPS-induced inflammatory PTB model, which may not fully recapitulate the multifactorial etiology of human PTB, including cases driven by non-infectious causes such as stress or vascular disorders.

In conclusion, this study identifies the RIPK1/RIPK3-MLKL pathway as a critical mediator of inflammation and necroptosis in PTB. Through comprehensive in vitro and in vivo experiments, we have demonstrated that RIPK1 activation drives both inflammatory responses and trophoblast death, and that targeting this pathway can effectively mitigate these pathological processes. These findings not only advance our understanding of PTB pathogenesis but also identify promising therapeutic strategies for this clinically significant condition.

## Methods

### Cell culture and treatment

HTR8/SVneo cells, a human trophoblast cell line obtained from ATCC (Manassas, VA, USA), were maintained in Dulbecco’s modified Eagle’s medium (DMEM)/F12 (ATCC) supplemented with 10% fetal bovine serum (FBS; ATCC) and 100 IU/mL penicillin-streptomycin (ATCC) at 37 °C in a humidified atmosphere containing 5% CO₂. The cell line was authenticated by short tandem repeat (STR) profiling upon acquisition and was routinely tested negative for mycoplasma contamination using a qPCR-based assay prior to key experiments. Upon reaching 80% confluence, the cells were washed with PBS and serum-starved in FBS-free DMEM/F12 for 12 h. Subsequently, they were treated with recombinant human TNF-α (10 ng/mL) in complete medium for 24 h or 48 h. For inhibitor studies, cells were pretreated for 1 h with either 10 μM necrostatin-1 (Merck Millipore, Darmstadt, Germany) or 10 μM GSK2982772 (Cayman Chemical, Ann Arbor, MI, USA) prior to TNF-α stimulation.

### Cell transfection

Plasmids encoding short hairpin (sh) RNA targeting RIPK1 (shRIPK1) and a non-targeting negative control (shNC) were purchased from Shanghai GenePharma Company. HTR8/SVneo cells were transfected with these plasmids using Lipofectamine 3000 (Invitrogen, Carlsbad, CA, USA) according to the manufacturer’s protocol. Following transfection, cells were subjected to treatment with recombinant human TNF-α as described above.

### Quantitative real-time PCR (qPCR)

Total RNA was isolated from HTR8/SVneo cells using TRIzol reagent (Invitrogen), and the concentration and purity of the extracted RNA were assessed using a NanoDrop spectrophotometer (Thermo Fisher Scientific, Waltham, MA, USA). First-strand cDNA was synthesized from 1 μg of total RNA using the PrimeScript RT Reagent Kit (Takara Bio, Kusatsu, Shiga, Japan) according to the manufacturer’s instructions. The resulting cDNA was subsequently used as a template for qPCR amplification in a 20 μL reaction mixture containing 2× SYBR Green PCR Master Mix (Applied Biosystems, Foster City, CA, USA). Amplification was performed on a QuantStudio 6 Flex Real-Time PCR System (Applied Biosystems). The relative mRNA expression of RIPK1 was normalized to GAPDH as an endogenous control and calculated using the 2^−ΔΔCt^ method.

### Western blot

HTR8/SVneo cells were lysed in RIPA buffer (Thermo Fisher Scientific) supplemented with protease and phosphatase inhibitors (Roche, Basel, Switzerland). The lysates were then clarified by centrifugation at 12,000 × g for 15 min at 4 °C, and the resulting supernatants were collected. Protein concentration was subsequently determined using a BCA assay kit (Pierce Biotechnology, Rockford, IL, USA). Equal amounts of protein (20 μg) were resolved by 10% SDS-PAGE (Bio-Rad Laboratories, Hercules, CA, USA) and transferred to PVDF membranes (MilliporeSigma, Burlington, MA, USA). The membranes were then blocked with 5% non-fat milk (Bio-Rad) in TBST for 1 h at room temperature. Subsequently, the membranes were incubated overnight at 4 °C with primary antibodies against RIPK1 (73271S; CST, Danvers, MA, USA), phospho-RIPK1 (Ser166) (44590S; CST), phospho-RIPK3 (Thr231/Ser232) (91702S; CST), phospho-MLKL (Ser345) (37333S; CST), Cleaved Caspase-3 (Asp175) (9664S; CST), Cleaved Caspase-1 (Asp296) (89332S; CST), and GSDMD-N (N-term) (39754S; CST). Following several washes with TBST, the membranes were incubated with HRP-conjugated secondary antibodies (1:5000; CST) for 1 h at room temperature. Immunoreactive bands were visualized using ECL substrate and imaged with a ChemiDoc system (Bio-Rad), with GAPDH (5174S; CST) serving as a loading control.

### Enzyme-linked immunosorbent assay (ELISA) and assay kit

Conditioned media were collected from stimulated HTR8/SVneo cells by centrifugation (1000 × g for 10 min at 4 °C), while mouse serum was clarified by centrifugation (2000 × g for 15 min at 4 °C). All samples were stored at −80 °C until analysis. The concentrations of various cytokines (IL-1β, IL-6, TNF-α) and uric acid were quantified using the following commercial ELISA kits: human/mouse IL-1β (ab214025/ab197742, Abcam, Cambridge, MA, USA), human/mouse IL-6 (ab178013/ab222503, Abcam), human/mouse TNF-α (ab181421/ab208348, Abcam), and uric acid (ab65344, Abcam). All assays were performed according to the manufacturers’ instructions. Briefly, standards and samples (50–100 μL/well) were added to pre-coated 96-well plates and incubated for 2 h at room temperature (RT). After washing, the plates were incubated with detection antibodies (1 h, RT), followed by HRP-streptavidin (30 min, RT). Color was then developed using a TMB substrate, and the reaction was terminated by the addition of 1 N H₂SO₄. Absorbance was subsequently measured at 450 nm on a BioTek microplate reader.

### Propidium Iodide (PI) staining

Cell death in HTR8/SVneo trophoblasts was assessed using PI staining. After treatment, cells were collected and incubated with 10 μL PI (Vazyme Biotech, Jiangsu, China) reagent for 10 min. Images of multiple cell fields were captured using a fluorescence microscope immediately after staining, and the percentage of PI-positive cells was calculated.

### Animal research

All animal procedures were performed in accordance with institutional guidelines and approved by the Animal Ethics Committee of People’s Hospital of Rizhao. Female BALB/c mice (6−8 weeks old, 20−25 g body weight) were purchased from The Jackson Laboratory (Bar Harbor, Maine, USA) and housed and maintained under controlled conditions (12 h light/dark cycle, 23−25 °C) with ad libitum access to food and water. Following one week of acclimatization, female mice were mated by co-caging with male mice overnight starting at 20:00. The presence of a vaginal plug at 8:00 the next morning was considered evidence of successful mating, with this timepoint designated as embryonic day 0.5 (E0.5). The pregnant mice were then randomly assigned to one of four experimental groups (*n* = 6 per group) using a computer-generated random number sequence: control (vehicle only), LPS (lipopolysaccharide challenge), Nec-1 (necrostatin-1 treatment), and LPS+Nec-1 (co-treatment). The randomization was performed by an investigator not involved in the subsequent treatments or outcome assessments to avoid selection bias. On E15.5, mice in the respective groups received intraperitoneal (i.p.) injections every 12 h as follows: the LPS group received 0.5 mg/kg Escherichia coli O111:B4 LPS (Sigma-Aldrich, St. Louis, MO, USA) in 100 μL sterile normal saline (NS); the Nec-1 group received 0.5 mg/kg Nec-1 (Sigma-Aldrich) in 100 μL NS; the LPS+Nec-1 group received both agents at the same doses; and the control group received an equivalent volume of NS. Following the initial LPS administration, mice were monitored every 2 h for labor onset. Upon parturition or at the predetermined endpoint (8 h post-injection for control mice), mice were euthanized via 5% isoflurane inhalation. Placental tissues were then rapidly harvested. For Western blot and ELISA analysis, placental tissues were snap-frozen in liquid nitrogen and stored at −80 °C. The sample size was also estimated to ensure adequate statistical power (≥80%) to detect a pre-specified effect size (α = 0.05, β = 0.20) using G*Power software, based on preliminary data showing a significant difference in cytokine levels and necroptosis markers between control and LPS-treated groups. Investigators involved in treatment, outcome assessment, and data analysis were blinded to group assignments throughout the study.

### HE staining

Placental tissues were fixed in 10% neutral buffered formalin for 48 h, dehydrated through graded ethanol (70–100%), cleared in xylene, and embedded in paraffin. The paraffin blocks were then sectioned at a thickness of 5 μm using a Leica RM2235 microtome and mounted onto poly-L-lysine-coated slides. For staining, sections were first deparaffinized in xylene and rehydrated through a descending series of ethanol concentrations. The sections were then stained with Harris hematoxylin (Thermo Fisher Scientific) for 5 min, followed by a brief rinse in water. Differentiation was performed in 1% acid alcohol, after which the sections were blued in 0.2% ammonia water for 30 sec. Subsequently, they were counterstained with eosin Y (Thermo Fisher Scientific) for 2 min and dehydrated through an ascending series of ethanol concentrations before xylene clearing. Finally, sections were mounted with Permount and imaged using a Nikon Eclipse Ci microscope equipped with a DS-Fi3 camera.

### Statistical analysis

Animals or samples were excluded from the analysis only in cases of technical failure (e.g., tissue degradation during processing, or failure in RNA/protein extraction). These exclusion criteria were pre-established prior to data collection. No animals were excluded post-allocation based on outcome measures.

All data are presented as the mean ± standard deviation (SD). The sample size (*n*) for each experiment is provided in the figure legends and represents biological replicates. Statistical analysis was performed using GraphPad Prism 7. Prior to statistical comparisons, the normality of data distribution was confirmed using the Shapiro-Wilk test. The homogeneity of variances between groups was verified using Brown-Forsythe test. For comparisons between two groups, an unpaired Student’s *t*-test was used. For comparisons among multiple groups, one-way analysis of variance (ANOVA) was employed, followed by Tukey’s post hoc test for multiple comparisons. The specific statistical tests used were justified as appropriate for the experimental design and the confirmed characteristics of the data. A *p*-value of less than 0.05 was considered statistically significant.

## Supplementary information

Western blot images

Supplementary Figure 1

## Data Availability

The datasets used and/or analysed during the current study are available from the corresponding author on reasonable request.
