# Supplementary figures and images for: Inhibition of RIPK1/RIPK3-MLKL inflammatory signaling pathway activation attenuates preterm birth
Source: Cell Death Discov. 2026 Apr 18;12:255. doi: 10.1038/s41420-026-03093-z (PMC13219625; doi:10.1038/s41420-026-03093-z)

Figure 1C+S1E


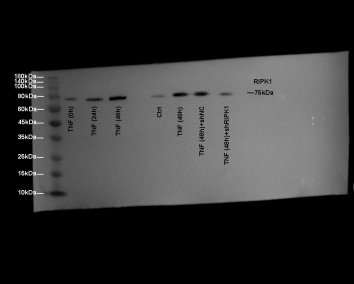

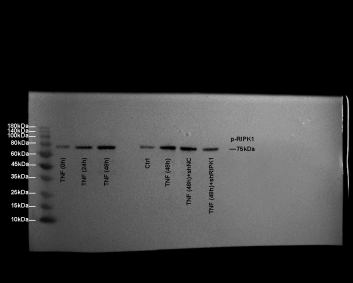

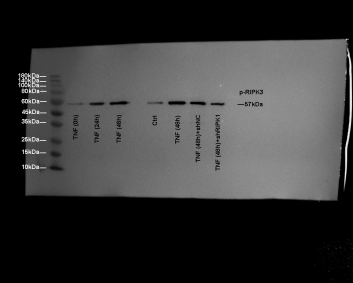


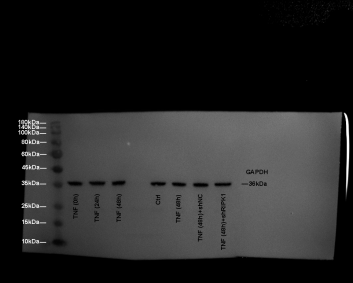

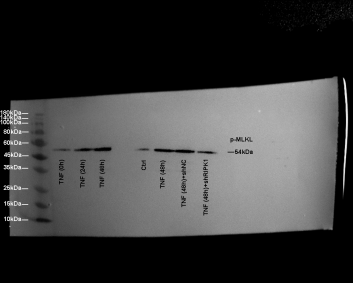


Figure 1D+2F


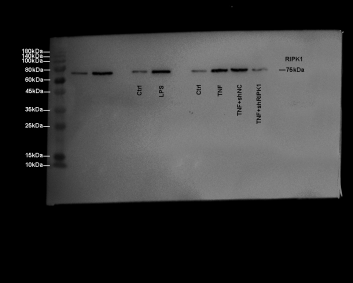

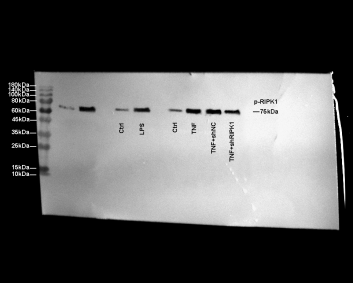

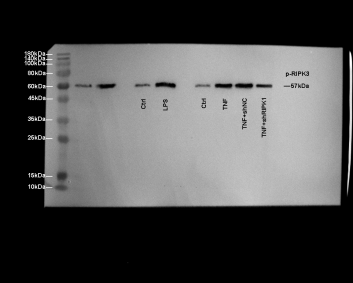


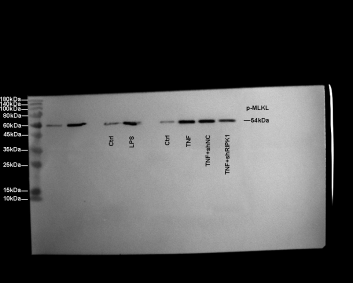

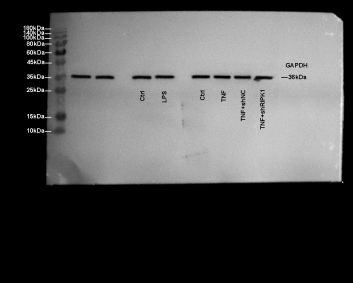


Figure 1E+1F


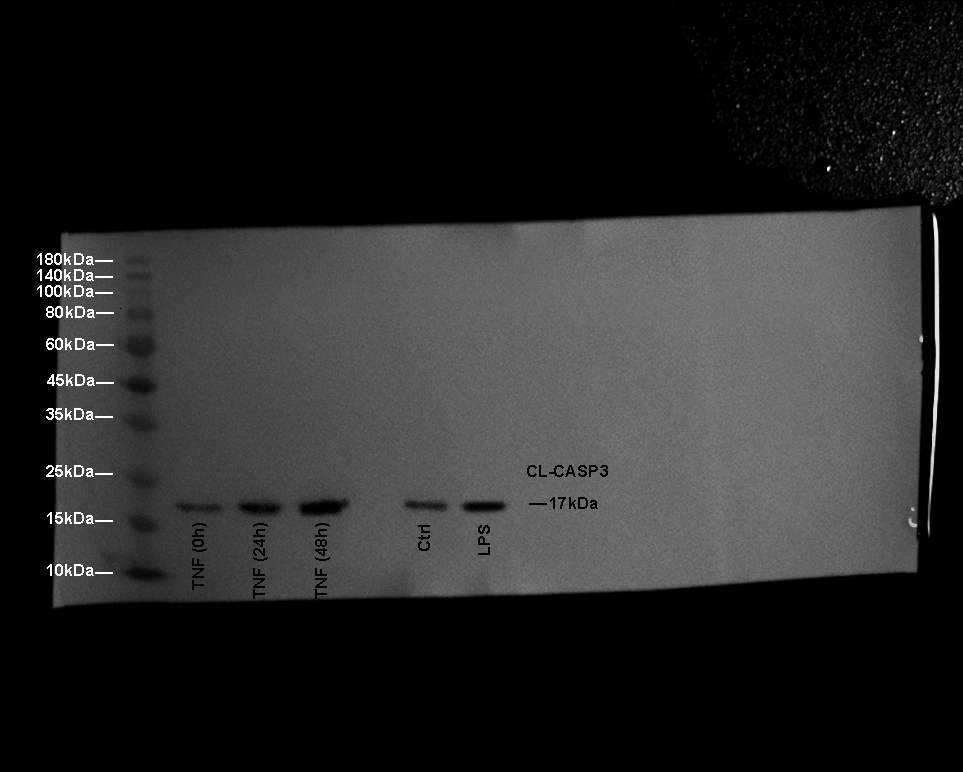

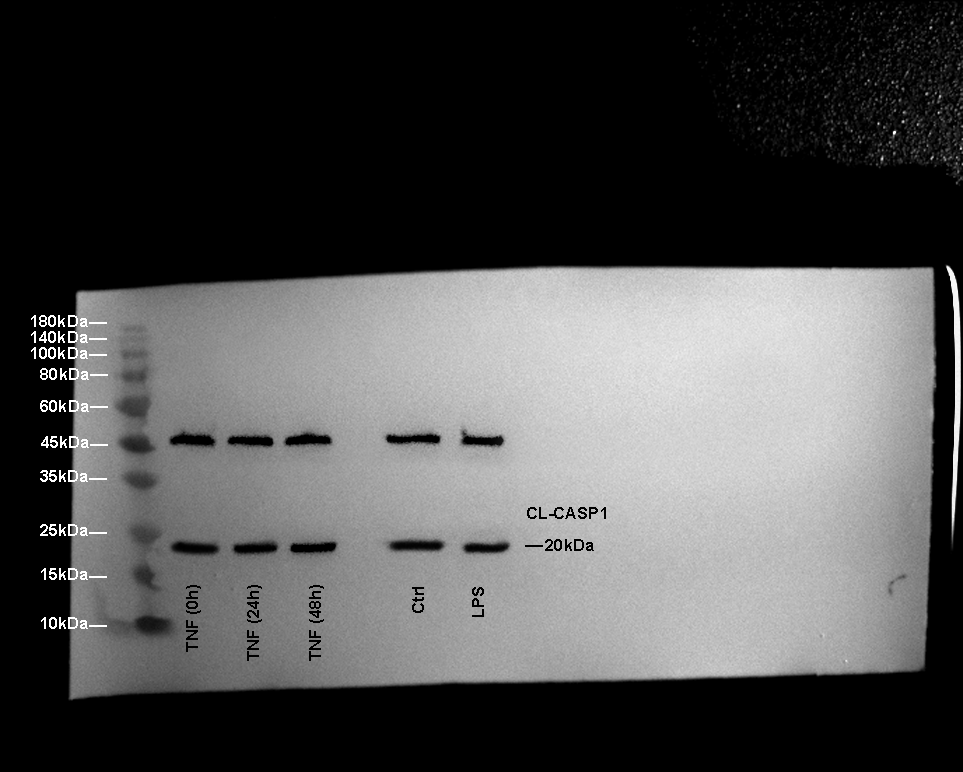

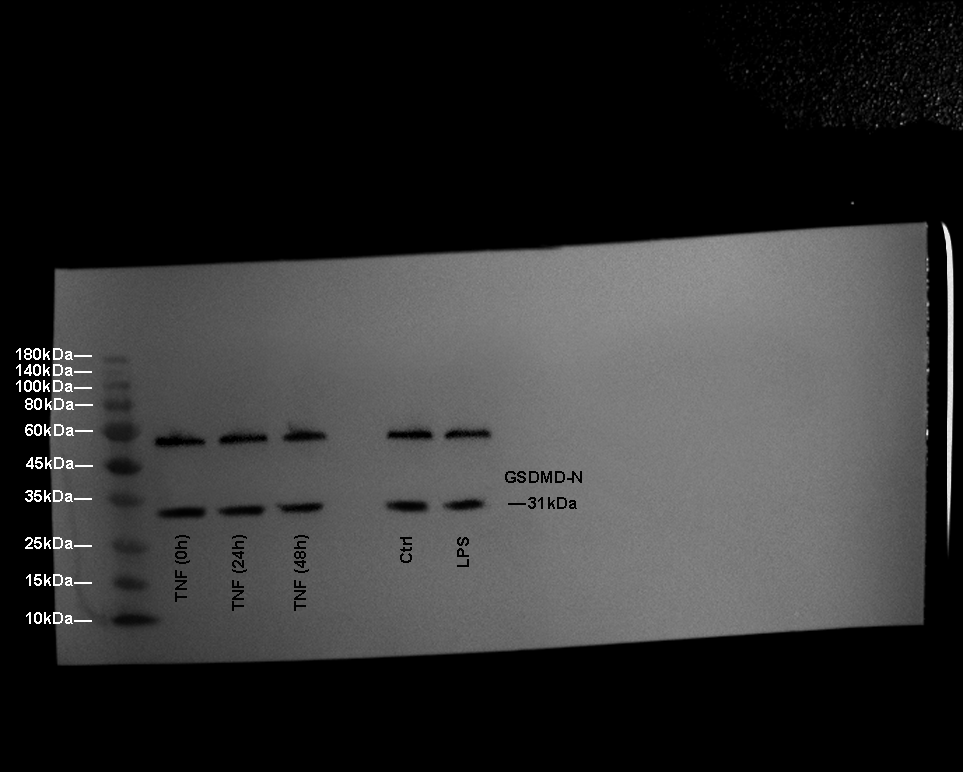


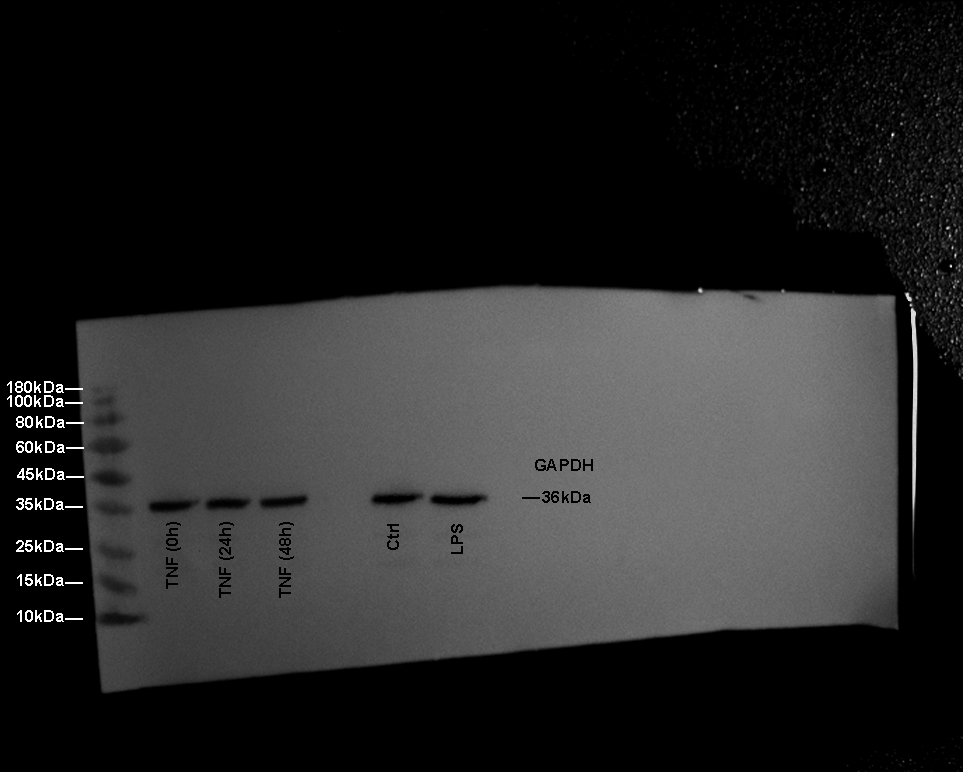


Figure 3E+4E+5E


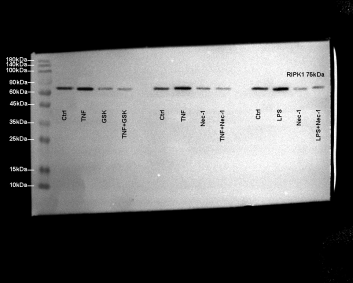

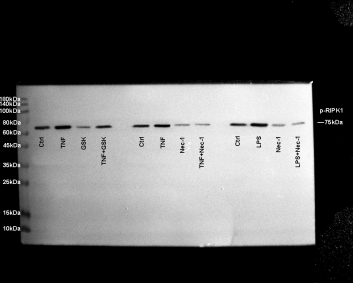

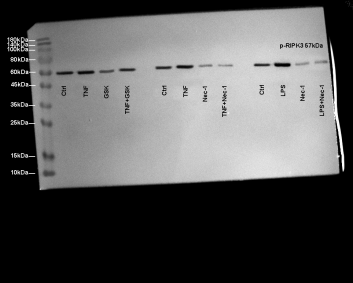


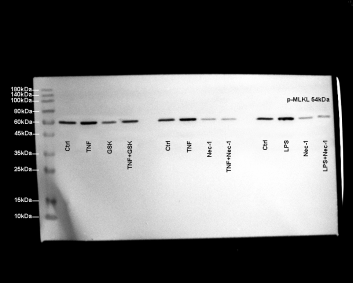

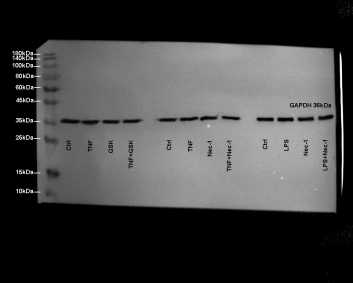

Supplement: Supplementary file 1 — Western blot images [file 41420_2026_3093_MOESM1_ESM.docx]

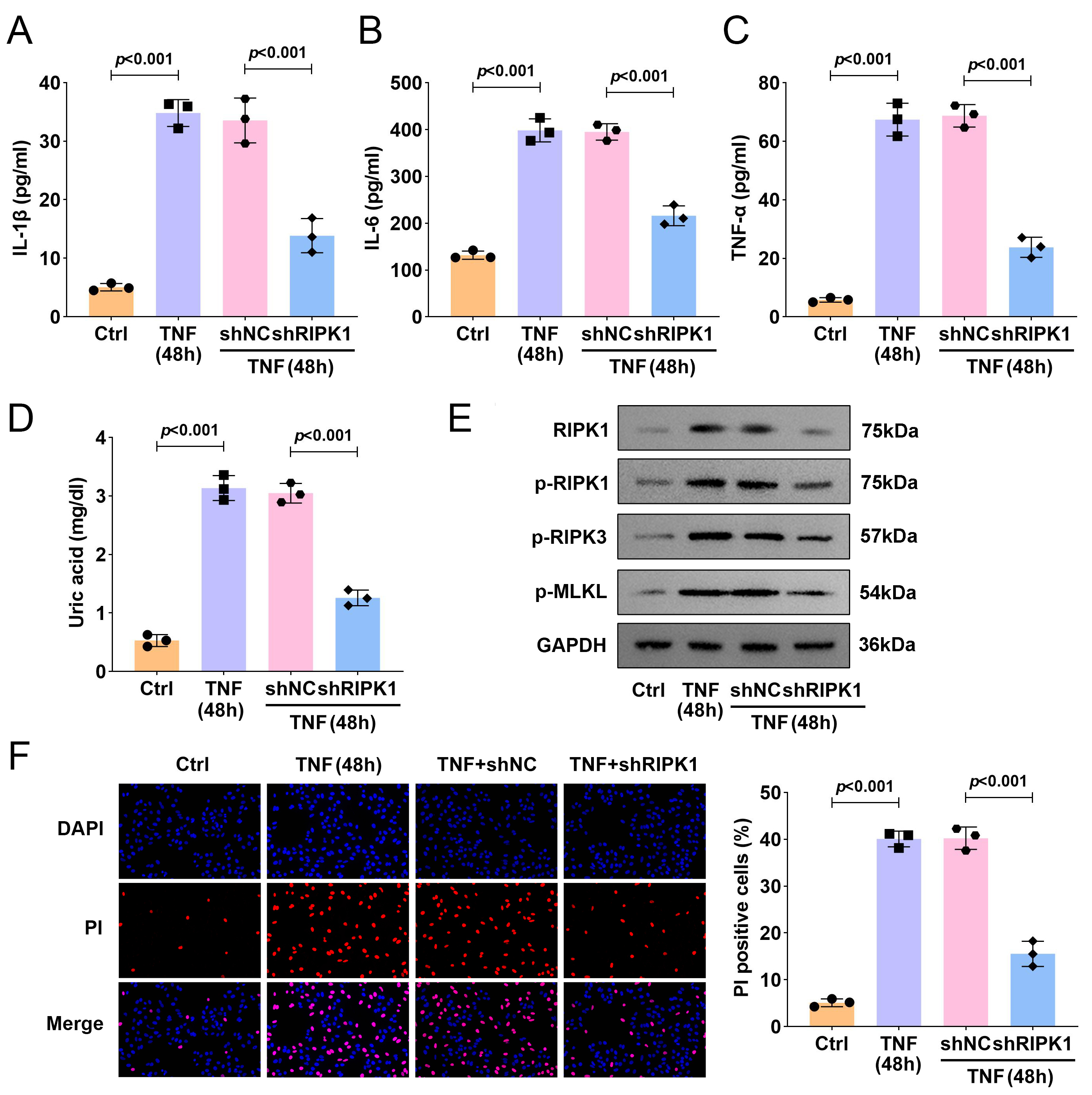

Supplement: Supplementary file 2 — Supplementary Figure 1 [file 41420_2026_3093_MOESM2_ESM.jpg]
